# Socio-affective touch expression database

**DOI:** 10.1371/journal.pone.0190921

**Published:** 2018-01-24

**Authors:** Haemy Lee Masson, Hans Op de Beeck

**Affiliations:** Department of Brain and Cognition, KU Leuven, Leuven, Belgium; Boston Children's Hospital / Harvard Medical School, UNITED STATES

## Abstract

Socio-affective touch communication conveys a vast amount of information about emotions and intentions in social contexts. In spite of the complexity of the socio-affective touch expressions we use daily, previous studies addressed only a few aspects of social touch mainly focusing on hedonics, such as stroking, leaving a wide range of social touch behaviour unexplored. To overcome this limit, we present the Socio-Affective Touch Expression Database (SATED), which includes a large range of dynamic interpersonal socio-affective touch events varying in valence and arousal. The original database contained 26 different social touch expressions each performed by three actor pairs. To validate each touch expression, we conducted two behavioural experiments investigating perceived naturalness and affective values. Based on the rated naturalness and valence, 13 socio-affective touch expressions along with 12 corresponding non-social touch events were selected as a complete set, achieving 75 video clips in total. Moreover, we quantified motion energy for each touch expression to investigate its intrinsic correlations with perceived affective values and its similarity among actor- and action-pairs. As a result, the touch expression database is not only systematically defined and well-controlled, but also spontaneous and natural, while eliciting clear affective responses. This database will allow a fine-grained investigation of complex interpersonal socio-affective touch in the realm of social psychology and neuroscience along with potential application areas in affective computing and neighbouring fields.

## Introduction

Humans frequently express emotions and intentions through nonverbal communication [1]. Among these nonverbal communication behaviours, facial expression has received the most attention, yielding more than a hundred different facial expression databases (e.g., GEMEP Corpus [2] and MMI Facial Expression Database [3]). Yet, interpersonal touch also conveys a vast amount of information such as socio-affective state and physical and psychological closeness between the interacting people [4]. Thus, an expression through touch is a potent and hard-to-ignore means of conveying a message, notwithstanding the individual and cultural variation in terms of how, how often, and with whom, touch communication is employed. Moreover, interpersonal touch often has a strong emotional valence. Positive affective touch plays an important role in making the receiver feel support, reassurance, affection or sexual attraction [5]. In contrast, negative affective touch can convey anger, frustration or disappointment [6]. Over the past decade, psychologists and neuroscientists have increasingly studied the role of touch in social context, how the emotions were conveyed through touch, and its neural basis [4, 7]. Despite of the importance and high frequency of touch communication and increasing interest in this topic, however, stimuli created for these studies poorly represent the actual phenomenon of socio-affective touch, and are mostly limited to video clips showing simple events such as a hand being stroked, slapped or brushed [8–15]. These studies therefore only address a very small part of "affective touch" since humans use much more complex and varied interactions to express affective intentions (both positive and negative) by touch. A wider range of more complex interpersonal affective touch events has so far only been addressed in studies using static images (for example, using pictures of kissing, hugging, and hitting as stimuli: [16–19]. However, there has been no attempt to implement complex dynamic affective touch stimuli in this domain. Crucially, none of the aforementioned studies varies their stimuli for levels of different valence and arousal, leaving important evaluative dimensions open [20].

To allow for more rigorous study of the social and affective impact of touch, we developed the Socio-Affective Touch Expression Database (SATED), with the aim to cover a larger range of dynamic interpersonal socio-affective touch actions, which span two primary affective dimensions, valence and arousal. To reach this general aim, we started by designing systematically defined, well-controlled socio-affective touch expression stimuli. Next, we validated the affective values and characterised the physical aspects of these stimuli using a behavioural experiment and a computer vision algorithm respectively. Lastly, we created additional video clips containing situations in which objects were touched without inducing specific emotions (neutral touch towards objects) while the movements used for object-based touch (non-social) itself were matched with the movements used in interpersonal socio-affective touch.

## Materials and methods

### Creation of a stimulus set of socio-affective and non-social touch videos

The major goal of the current study is to create and validate dynamic interpersonal socio-affective touch video clips that span a large range of touch communication events, as well as their corresponding object-based non-social touch events. In order to cover two primary affective dimensions, valence and arousal, we included negative, neutral, and positive touch situations, which are associated with low (calm) to high (exciting) arousal. We included available touch communications reported in different studies that involve the human body such as “hug” and “pat” [18, 19, 21]. Although we tried to incorporate a sufficiently wide spectrum of touch communications in our database, we do not claim that our list is exhaustive. After deciding which touch communications to include, we imagined and created scenarios that would evoke a certain touch communication. Examples of negative situations include punching and pushing a person on his/her arm. Examples of positive situations include different forms of gently stroking a person (for the purpose of consolation or flirting) and hugging. Examples of neutral situations include shaking hands as a greetings and nudging the arm to get someone’s attention.

Six actors (three actor-pairs, age = around 35), consisting of pairs of friends/close colleagues from KU Leuven, without any professional acting experience volunteered in the recordings. The idea behind recruiting non-professional actors was to capture spontaneous and potentially more life-like touch expressions rather than those obtained from trained prototypical acting. To the best of our knowledge, no study so far has compared the differences between touch communication databases performed by a professional actor and a non-professional ordinary person. In the context of recording facial expression performances, however, Kaulard and her colleagues have emphasized the negative aspects of recruiting professional actors due to their exaggerated, caricature-like, stereo-typed performances [22]. Each actor pair consisted of one male and one female actor who were familiar to each other, and between whom at least neutral touch expressions had been frequently used in a real life situation. Therefore, actors did not feel awkward to use touch communication during recordings. On the day of recordings, actors wore either black or grey long-sleeved shirts so as not to induce biased reactions towards the clothing styles between actors while also providing a high visual contrast for distinguishing the actors from each other and the background. All the videos were recorded in the same place where an empty white wall was used as a background. The camera used for recordings had a resolution of 1280 (width) × 720 (height) pixels with a frame rate of 29 frames per second. The camera was mounted to a tripod at a distance of 1.71 metres from the actors.

Each pair of actors performed 26 different socio-affective touch scenes in front of the camera. Both actors in the pair took a turn of performing as a touch initiator and a receiver during the practice and decided how to best select the role. When the actors were not able to decide, we filmed two scenes per scenario and decided which one to take when editing the videos. A method-acting protocol was used in which actors were instructed to read a scenario description provided beforehand and to think about a similar situation in their life in order to call up the emotions and actions that one needs in the filming situation [23]. Here, we asked actors to use a specific action (hug, stroke, hit or shake) and duration (approximately three seconds) for each touch expression. In this way, we could still control the action and duration while making other factors such as speed and pressure of the touch as natural as possible.

Table 1 lists the scenarios describing the situations that evoke intended touch expressions in the final stimulus set. This list only contains a basic set of stimuli that were retained after the validation test (See results) and includes both socio-affective and corresponding object-based touch events. It contains 13 socio-affective events x 3 actor-pairs, and in addition 12 object-based touch events. Full scenarios of the original larger set of socio-affective touch events (26 events × 3 actor-pairs) can be found in S1 Table.

**Table 1:** Scenarios of the final set of touch events.

Video Number	Scenario	Valence	Action	Stimulus Name
**1, 14, 27**	You are in the airport. You and your partner have not seen each other for 6 month. You hug each other as soon as you see each other.	Positive	Hug	Hug1_p
**2, 15, 28**	Your partner looks somehow lovelier today. You want to give a hug to express how much you love this person.	Positive	Hug	Hug2_p
**3, 16, 29**	Your sibling just ended the long-term relationship. You want to console him (her) by hugging.	Positive	Hug	Hug3_p
**4, 17, 30**	You want to flirt with him (her) by stroking his (her) arm with intimacy.	Positive	Stroke	Str1_p
**5, 18, 31**	Your friend is crying. You want to console this person by stroking on his (her) arms.	Positive	Stroke	Str2_p
**6, 19, 32**	You hold hands of your partner with affection.	Positive	Hold	Hold1_p
**7, 20, 33**	You want to get an attention from your colleague (who could not hear you calling) by tapping his (her) shoulder.	Neutral	Tap	Tap1_neu
**8, 21, 34**	You just found out that your partner cheated on you. You are very disappointed and angry. You ask him (her) how and why this happened aggressively while shaking his (her) whole body.	Negative	Shake	Sha1_n
**9, 22, 35**	Your sibling committed crime. You are sad and angry since you cannot understand why he (she) made such a horrible decision. You firmly ask him (her) how and why this has happened while shaking his (her) arm.	Negative	Shake	Sha2_n
**10,23,36**	Your partner said that you two are over from now on. He or she is about to walk away and never see you again. You cannot let this person go. You grip and pull this person’s arm desperately.	Negative	Grip	Gri1_n
**11,24,37**	You are in the metro. You need to get off from the metro. There is somebody blocking you. You nudge your way by slightly removing somebody’s arm blocking you.	Negative	Nudge with elbow	Nudg1_n
**12,25,38**	Your sibling tries to be silly by making a weird gesture. You are annoyed. You want to stop him (her) by poking in the ribs.	Negative	Nudge with elbow	Nudg2_n
**13,26,39**	Your sibling always makes the same mistakes. You slap his (her) arm to make him (her) realize that you are annoyed and that you expect something better.	Negative	Slap	Slap1_n
**40,52,64**	Carry the water bottle.	Neutral	Hug	Hug2_ob
**41,53,65**	Carry the big box.	Neutral	Hug	Hug3_ob
**42,54,66**	Examine the texture.	Neutral	Stroke	Str1_ob
**43,55,67**	Remove possible winkles from the suit jacket hung on the hanger.	Neutral	Stroke	Str2_ob
**44,56,68**	Carry the basket.	Neutral	Hold	Hold1_ob
**45,57,69**	Ring the desk bell to get attention.	Neutral	Tap	Tap1_ob
**46,58,70**	Empty the dust from the plastic box.	Neutral	Shake	Sha1_ob
**47,59,71**	Shake the cocktail in the mixer.	Neutral	Shake	Sha2_ob
**48,60,72**	Grip and shake the Champaign bottle before opening it.	Neutral	Grip	Gri1_ob
**49,61,73**	Pass by a door while elbowing it closed.	Neutral	Nudge	Nudg1_ob
**50,62,74**	Push with arm to close the door.	Neutral	Nudge	Nudg2_ob
**51,63,75**	Remove dust from the carpet.	Neutral	Slap	Slap1_ob

This table lists the scenarios of the final set of touch events. Video number illustrates the file name of each video uploaded (basic version). The expected valence and used action are described along with the corresponding stimuli name for each touch expression. The order in which the touch events were recorded was according to the order written in this table.

For the object-based touch events that cover the non-social touch category, we included situations where similar motions were required to interact with a particular object. For example, the touch scene in which the fabric of clothing is stroked was included as a motion match to the touch event where one actor strokes the other’s arm. It should be taken into account that we created object-based touch scenarios after the validation test on social stimuli to match the action between social and non-social video clips. The idea behind having object-based touch videos, as a set of non-social stimuli, is that such a contrast condition might be useful for various experimental applications, such as neuroimaging and neuropsychological experiments. Especially, when investigating the valence and arousal differences between social and non-social touch conditions, confounding effects such as hand motions shown in touch events can be ruled out. Therefore, actors returned back to perform the object-based touch events on a later date. They were instructed to wear the same clothes that they used for the social-affective touch events. Care was taken that the object-based touch scenarios would not induce distinctive emotional responses, hence expected to be “relatively neutral” in terms of valence ratings.

**Fig 1:**
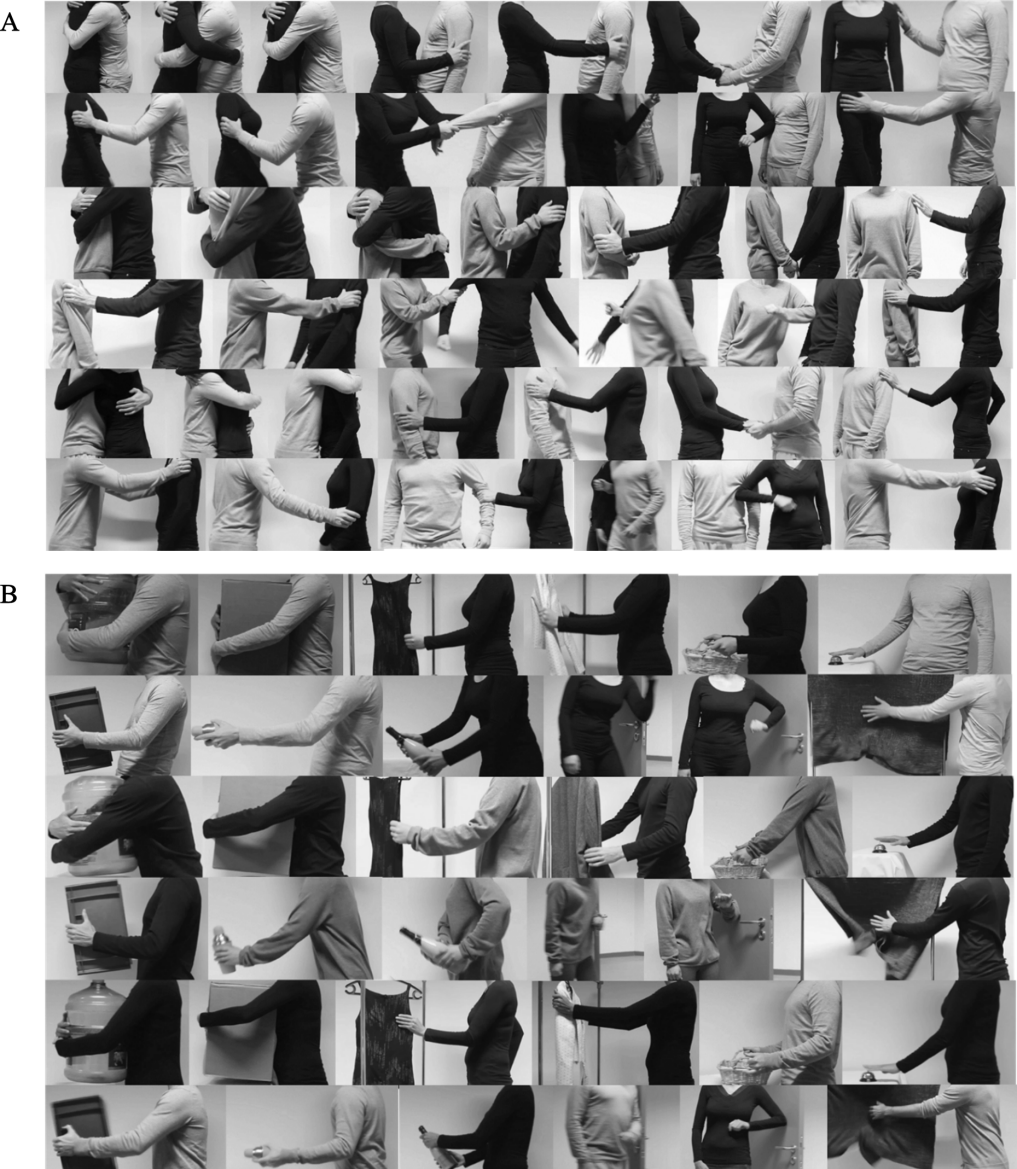
Socio-affective and non-social touch stimuli. A) The figure shows representative still frames from the basic set of socio-affective stimuli, showing different types of interpersonal touch events (positive (the first six stimuli in the 1st, the 3rd and the 5th rows), neutral (the last stimuli in the 1st, the 3rd and the 5th rows) and negative events (six stimuli in the 2nd, the 4th and the 6th rows)). B) The figure shows representative still frames from the matched object-based non-social stimuli, showing different interactions with various objects. The touch expressions are available as dynamic video clips.

For all touch events, actors were allowed to practice the event beforehand until it felt natural to perform. Once they agreed to start, they performed each scene after hearing the voice of the director who recorded the video clips saying, “start”. During recordings, actors were allowed to produce facial and verbal expressions if necessary in order to keep the naturalness. Both audio and face information were discarded during the recording and the editing, with the focus being only on the movement of torso. Moreover, in the case of two videos filmed for one scenario, a director (who filmed the videos) and the author discussed and made a decision which one to keep based on the naturalness of the performance (e.g. when it was evident that one video showed better naturalness over another) and usability of the video (e.g. when it was impossible to remove the face from the touch scene during editing or when the duration (average duration 3 seconds) of the video was too short (<1 seconds) or too long (> 5 seconds)). As a result, among the 78 videos in total, 36 videos show a male initiating the touch while 25 videos show a female initiating the touch. Both male and female acted the touch at the same time for 17 videos (e.g. shaking hands for the greeting). All actor-pairs were able to perform all touch actions in spite of different level of difficulty between stimulus events (e.g. neutral vs. aggressive expressions). Fig 1 shows representative still frames from the selected dynamic touch expressions.

### Validation of stimulus set

The purpose of this stimulus validation experiment was to test if the affective aspects of the social video clips are perceived as positive or negative as intended, while also measuring their naturalness so as to exclude artificially performed socio-affective touch scenes. To achieve these aims, we recruited participants who judged valence, arousal and naturalness of each stimulus (Experiment 1). After the selection of social touch stimuli was made, object-based touch stimuli were created and validated along with the social ones on valence and arousal ratings (Experiment 2). Affective responses to object-based touch events were examined to test whether object-based touch events are perceived as neutral events in contrast to social touch events. Lastly, the association between motion energy and affective characteristics were investigated.

#### Participants

First, 11 right-handed adults (6 female, mean age 27.2) rated the social video clips based on valence, arousal, and naturalness (Experiment 1). After selecting the optimized stimuli set through the validation test, 22 right-handed adults (10 female, mean age 26) were recruited to rate the valence and arousal for the final set of stimuli including both social and non-social video clips (Experiment 2).

All participants were randomly recruited through a participant database of laboratory of biological psychology online systems implemented in the KU Leuven webpage (https://ppw.kuleuven.be/lbp/questionnaires/lbp-fmri-experiment-questionnaire). Thus, we assume that participants were either (former) students or acquaintances of (former) students who knew the existence of KU Leuven and the webpage although we did not explicitly ask how they knew the webpage or their current professions. All participants are Europeans (mainly Belgians) living in Belgium when they took part in experiments. Importantly, no participant took part in both Experiment 1 and 2. All participants were naïve to the hypotheses of the study. Neither previous neurological nor psychiatric histories were reported among participants. Written informed consent was provided before the experiment. The study was approved by the ethical review board of KU Leuven (G-2016 06 569).

#### Stimuli and experimental design

In Experiment 1, the stimuli consist of video clips displaying interpersonal socio-affective touch scenes in the context of pleasant to unpleasant situations were validated during the experiment. The social stimuli included 26 scenes with positive (N = 10), neutral (N = 6) and negative (N = 10) touch interactions from three sets of actors (78 videos in total).

Before the experiment, the participants were instructed that they would watch series of videos containing touch events taking 3 seconds per item and that their task was to rate pleasantness (“How pleasant is the touch?”; 1-very unpleasant, 4-neutral, 7-very pleasant), arousal (“How arousing is the touch?”; 1-very calm to 7-very exciting) and naturalness (“How natural is the video clip?”; 1-very artificial, 4-neutral, 7-very natural) of the touch scene on a 7-point Likert scale using the keyboard. A few randomly chosen videos were shown as examples to the participants and the meaning of each scale was explained. Since some participants needed further explanations about the arousal scale, we emphasized that arousal referred to emotional arousal. Lastly, they were informed that there would be two sessions with the short break in between the sessions. Note that task instructions were given in English. This validation experiment would lead us to exclude stimuli with low naturalness ratings and to be able to evaluate if expected valence ratings were met the social stimuli.

In Experiment 2, object-based touch stimuli were created and validated together with the final set of 13 selected social scenarios. As mentioned above, the object-based touch stimuli were matched the actions of the social stimuli. Similar to the validation experiment described above, participants watched the videos, containing both socio-affective touch and object-based touch events, and evaluated them by rating its valence and arousal on a 9-point Likert-like scale using Self-Assessment Manikin (SAM) [24]: pleasantness (1-extremely unpleasant, 5-neutral, 9-extremely pleasant) and arousal (1-extremely calm to 9-extremly exciting). The same instructions were given to the participants in English.

**Fig 2:**
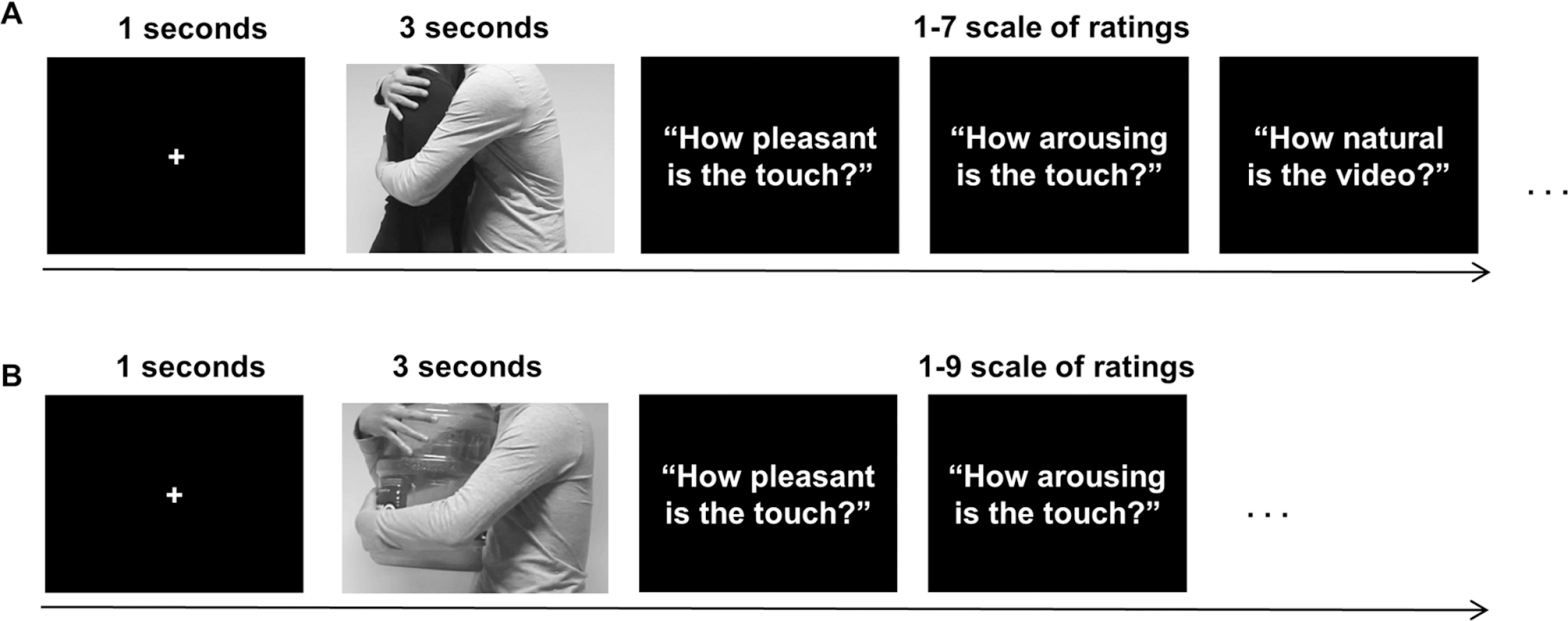
Experimental design. (A) The figure illustrates the experimental design for the validation test in Experiment 1. Participants were asked to answer three questions [valence, arousal and naturalness] using 1–7 scale after watching social touch videos for 3 seconds. (B) The figure shows how the object-based videos were validated along with the social videos in Experiment 2. Only valence and arousal were asked using 1–9 scale with SAM.

In both experiments, there were two sessions in which the same trials were repeated. The rating task was self-paced for each scale without posing a time-limit. Each session took approximately 15–20 minutes. After the first session, we provided a short break without posing a time-limit, all participants returned back to the second session within 10 minutes. Each video was shown once per session in a random order on a 23-inch LCD monitor (at a resolution of 640 (width) × 360 (height)) located at 0.5 metres from the participant. The experiment was controlled by Psychophysics Toolbox Version 3.0.12 (PTB-3) [25] in MATLAB. Fig 2 illustrates the procedures of the two experiments.

#### Analysis: Naturalness

Non-parametric statistical tests are implemented for the analyses due to observed violations of the normality assumptions in some sets of data (not reported). All statistical analyses were performed using the MATLAB (R2015a, The Mathworks, Natick, MA, USA) built-in functions such as signrank (Wilcoxon signed rank test for matched/paired data), ranksum (Mann-Whitney U test for two independent data), corr (Spearman correlation) and polyfit (second-degree polynomial regression).

In Experiment 1, for the naturalness test, we first averaged the ratings for each of the 26 scenarios (also referred to as stimuli) across all three actor-pairs and over both test sessions per participant. Then, we determined the group median on perceived naturalness for each stimulus.

In cases where a stimulus did not convey a sufficient level of naturalness, all three videos were to be excluded from further analysis. Our criterion for exclusion was to remove any stimuli with median ratings not significantly greater than 3 (slightly artificial), determined using Wilcoxon signed rank tests.

#### Analysis: Intra-, inter- subject and inter-actor consistency

In Experiment 1, using the Spearman rank-order correlation, we performed three consistency tests for each scale (valence, arousal and naturalness). Note that each participant rated 78 items (3 actor-pairs x 26 stimuli) in each test session, for a total of two sessions. We compared the ratings for each of the 25 scenarios (after removing an artificially perceived touch event, see the results part) between the two sessions (N_S_ = 2), participants (N_P_ = 11), and actor-pairs (N_AP_ = 3). Specifically, we aim to determine: 1) the consistency in ratings for each scenario between sessions for the same participant (intra-subject consistency) 2) the consistency between subjects in their ratings of the same scenario (averaged across both sessions) (inter-subject consistency) and 3) the consistency in ratings between different actor pairs over all videos (inter-actor consistency). Formally, the 3 consistency tests are: 1) intra-subject (measured across two sessions per participant, i.e. session variability), 2) inter-subject (pair-wise correlation across the pair of participants, i.e. between subject variability) and 3) inter-actor consistency (pair-wise correlation across actors performing the same scenario, i.e. actor variability).

Test A: Intra-subject consistency (S1 Fig)

1. Per participant, average the ratings for each of the 25 stimuli across all actor pairs (N_AP_ = 3), separately for each session (N_S_ = 2) (totalling 11 x 25 x 2 ratings per scale).
2. Correlate the ratings of 25 stimuli between the two test sessions for each participant (N_p_ = 11). As a result, we will have 11 correlation values.
3. Find the median correlation coefficient of these 11 correlation values.

Test B: Inter-subject consistency (S2 Fig)

1. Per participant, average the ratings for each of the 25 stimuli across all actor pairs (N_AP_ = 3) and across both sessions (N_S_ = 2) (totalling 11 x 25 ratings per scale).
2. Correlate the ratings (25 values per participant) between every pair of participants (producing a total of 55 comparisons).
3. Find the median correlation coefficient among the 55 correlation values.

Test C: Inter-actor consistency (S3 Fig)

1. For each of the 75 videos (3 actor-pairs x 25stimuli), average the ratings over all participants and both sessions (totalling 3 x 25 ratings per scale).
2. Correlate ratings between every pair of actor-pairs (producing a total of 3 comparisons).
3. Find the median correlation coefficient among 3 correlation coefficients.

#### Analysis: Affective responses

We performed second-degree polynomial regression between the group averaged median valence and arousal ratings in order to describe non-linear relationship between valence and arousal. Note that the fitted curve was used for the visualization to show whether our data exhibited a U-shaped distribution as previously reported [24]. Importantly, we evaluated if positive stimuli such as hugging were rated pleasantly and negative videos such as slapping unpleasantly. We also measured if object-based touch events induced distinctive emotional responses despite they were intended to be “neutral” in terms of valence ratings in order to use them in a non-social condition.

#### Analysis: The Spearman-Brown split-half reliability test

Additionally, we conducted reliability tests on rated valence, arousal and naturalness with a split-half correlational method that compares the ratings of one half of participants to the ratings of the second half. First, we averaged the ratings for each of the 25 stimuli across all actor pairs (N_AP_ = 3) and across both session (N_S_ = 2) for each participant. Afterwards, we randomly split the total group of participants into two sub-groups, followed by median averaging the individual ratings (25 values per participant) for each sub-group. As a result, two median averaged sub-group ratings were created for each scenario. Those two sub-group ratings were then correlated (the Spearman rank order correlation). Finally, we adjusted the resulting correlations with the Spearman-Brown split-half reliability coefficients formula to obtain an estimate of the reliability of the entire group per scale (valence, arousal and naturalness). We iterated this process 100 times, each time randomly designating participants into two sub-groups. The results from 100 iterations were averaged (medians). This median coefficient indicates the degree to which different observers give consistent ratings. Using the same methods, we also compared the sex differences in the ratings. At this time, we split the group into two sub-groups based on the sex (female vs. male) without iterations.

Note that the Spearman-Brown split-half reliability test is different from the inter-subject consistency test (see S2 Fig). The former is to test how the median ratings of each stimulus from two sub-groups are correlated after splitting the group into two halves while the latter is to test how the ratings from two individual participants are correlated when pairing the participants using every possible combination. The latter is relevant to access the reliability between individual participants, while the former provides an estimate of the reliability of the full dataset.

#### Analysis: Low level visual motion features

Physical parameters such as the amount and type of motion are intrinsically connected to the motion displayed in the videos. For example, slapping involves short transients of high-speed motion. Thus, identifying motion intensity enables us to understand its possible links with action and emotional responses. We identified the magnitude of the motion energies to characterize its interaction with affective properties of touch video clips. Moreover, we examined consistency across actor-pairs in terms of consumed total motion energies to identify the similarities among the actor-pairs while performing the touch expression in a quantitative manner. Lastly, we compared the motion intensity of socio-affective touch with those of object-based touch. An optical flow algorithm based on the Lucas-Kanade method [26, 27] was used that determines the flow field between two consecutive frames of dynamic stimuli under several constraints such as a type of smoothness of the flow field. Here, we used a derivative of a Gaussian filter as a temporal gradient filter suggested by Simoncelli and colleagues [28] (see also MATLAB built-in function opticalFlowLKDoG).

**Fig 3:**
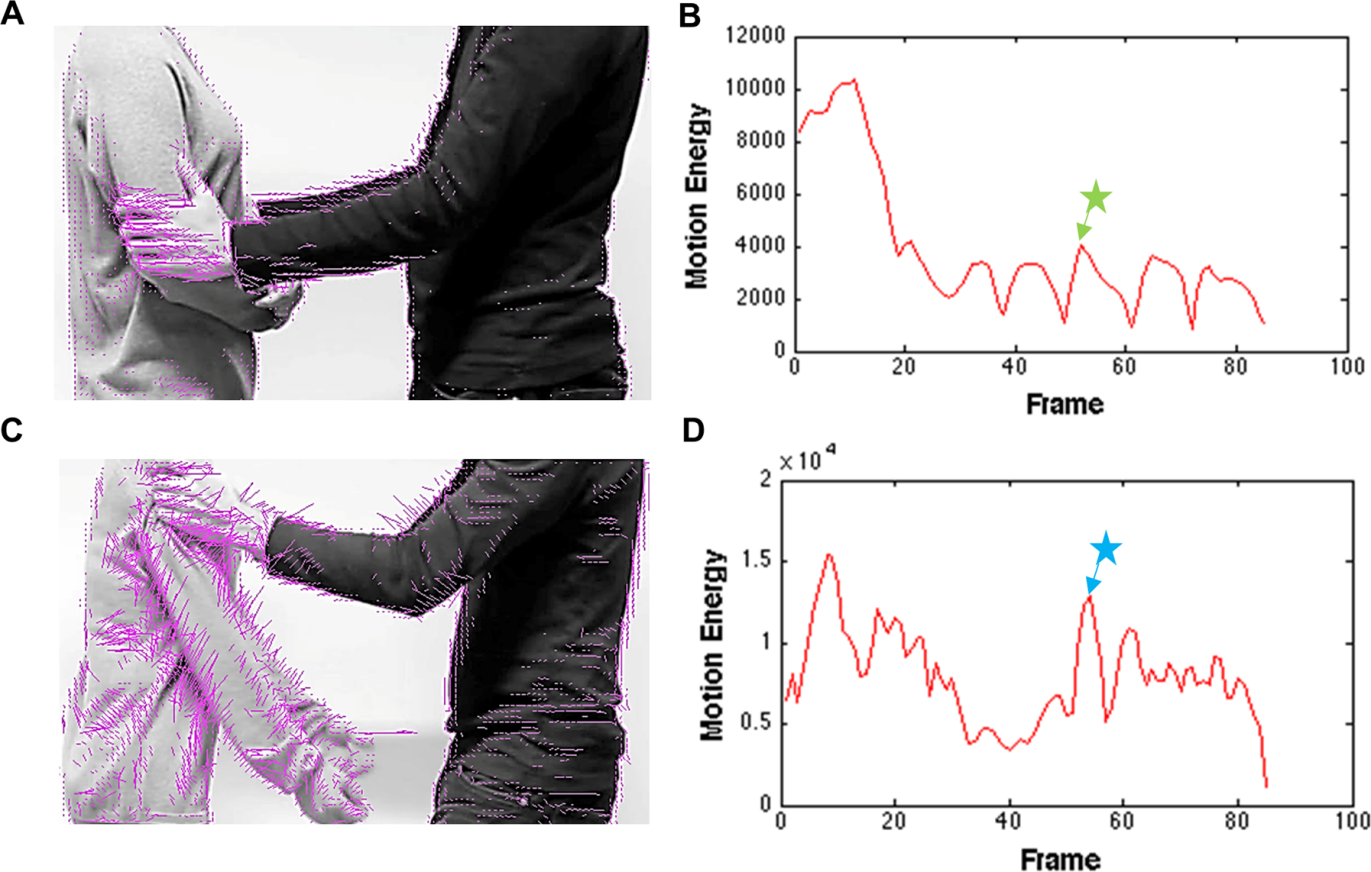
Motion energy. **(**A) and (C) show example frames from the video sequences of positive (A, stroking [stimulus “Str2_p”]) and negative (C, shaking [stimulus “Sha1_n”]) touch events with inserted pink lines. The length and the orientation of pink lines indicate speed and direction of the pixel movements respectively. (B) and (D) represent total motion energy of all pixels from the first frame to the last frame. The star signs with arrows in (B) and (D) indicate which frames are shown in (A) and (C) respectively.

With the algorithms, we obtained magnitude of motion energy by summing the absolute values of estimated velocities across the frames, which implies how much motion energy was consumed to perform particular actions (e.g. stroking or shaking) regardless of direction of the motion (Fig 3).

## Results

### Validation experiment

#### Experiment 1: Naturalness of the stimuli

**Fig 4:**
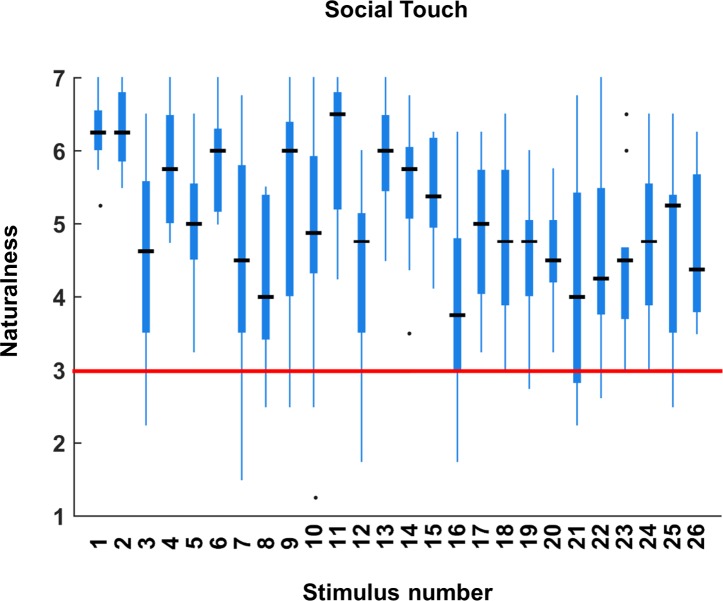
Perceived naturalness of stimuli. The figure demonstrates the individual naturalness ratings of 26 stimuli. The central black lines indicate the median naturalness ratings per scenario. The top and bottom edges of the boxes illustrate the 75th and 25th percentiles respectively while the whiskers show the range of the individual naturalness ratings. The black dots mean the outlying data points. The red line indicates the naturalness ratings 3 (slightly artificially perceived).

The results of the naturalness judgments indicated that the video clips were perceived naturally overall (median = 4.81, the mean absolute deviation (MAD) = 0.5). Fig 4 demonstrates individual naturalness ratings of each of the 26 scenarios, averaged across three actor-pairs and across two sessions. Based on the results, we excluded the stimuli rated rather artificially from the set of socio-affective stimuli and from the further analyses. Hence, stimulus number 16 (airport body checks) was excluded since the rated naturalness of these videos was not significantly greater than 3 (*z* = 43, *p* = 0.11) (See the red line in the Fig 4, see S1 Table for description of the stimulus). We also found a slight tendency that positive scenes (median = 5.38, MAD = 0.69) were perceived more naturally than the negative scenes (median = 4.63, MAD = 0.19) by observers (*z* = 1.94, *p* = 0.05).

#### Experiment 1: Intra-, inter- subject and inter-actor consistency

**Fig 5:**
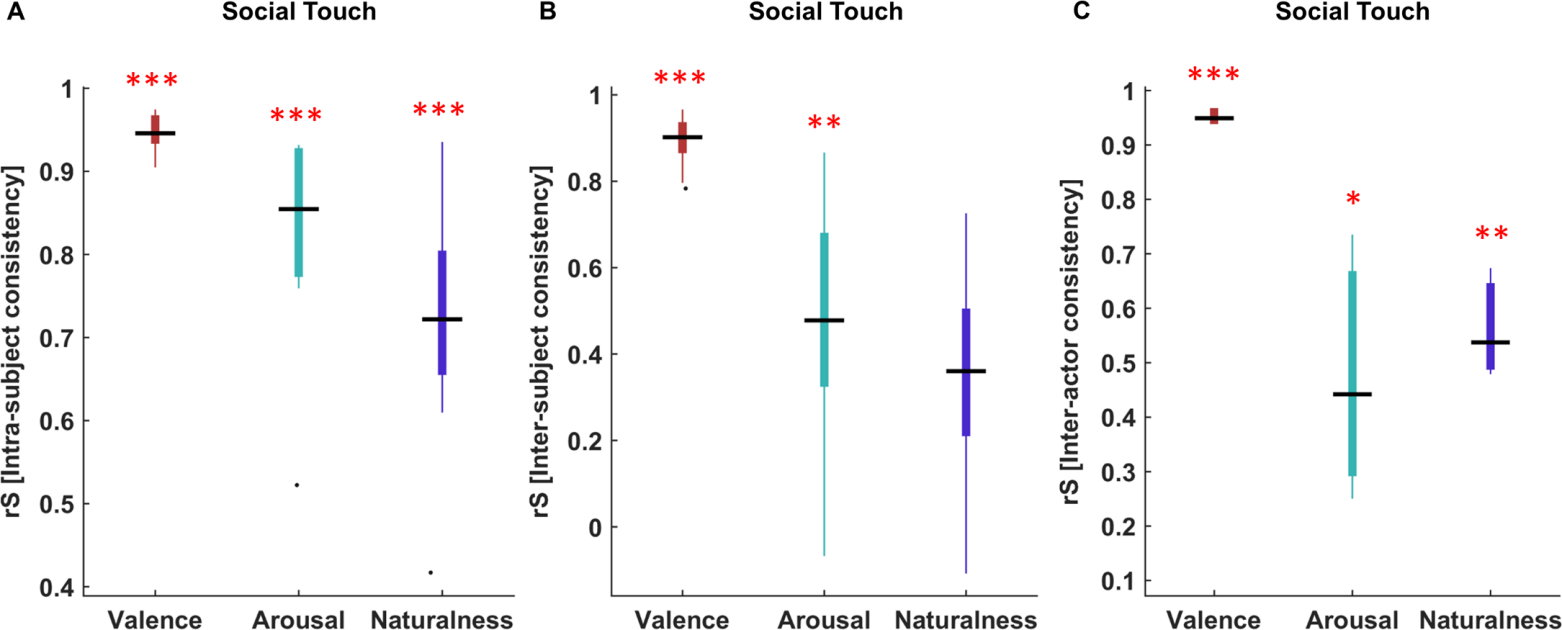
Intra-subject, inter-subject and inter-actor consistency. The figure illustrates the intra-subject consistency (A), the inter-subject consistency (B) and the inter-actor consistency (C) on valence (dark red), arousal (emerald) and naturalness (purple) ratings. In all figures, the central black lines indicate the medians of Spearman correlations. The top and bottom edges of the boxes illustrate the 75th and 25th percentiles respectively while the whiskers show the range of the rank correlation coefficients. The black dots indicate the outliers in (A) and (B). The single red asterisk indicates the statistical significance at *p* < 0.05, two red asterisks denotes statistical significance at *p* < 0.01, and three red asterisks show statistical significance at *p* < 0.001.

The results indicated that participants were consistent in their ratings between two sessions (valence median *rS* = 0.95 *p* < 0.001, range for individual participants 0.91–0.97; arousal median *rS* = 0.85 *p* < 0.001, range 0.52–0.93; naturalness median *rS* = 0.74 *p* < 0.001, range 0.42–0.93) (Fig 5A). Some outlying data points are observed (Fig 5A). However, the non-parametric statistics based upon medians rather than means should be robust to such outliers, and indeed removing outlying data points from the test did not change the results in a meaningful way (not reported).

Using a pair-wise comparisons method, we found high correlation of valence ratings between participants, suggesting high inter-observer consistency (median *rS* = 0.9 *p* < 0.001, range for individual participant pairings 0.78–0.96). In case of arousal (median *rS* = 0.48 *p* = 0.01, range -0.07–0.86), we found weaker correlations among participants. The pair-wise correlation of rated naturalness (median *rS* = 0.36 *p* = 0.08, range -0.11–0.72) across participants was not significant, however, implying larger individual variability for perceived naturalness (Fig 5B).

Lastly, we measured the inter-actor consistency to validate whether the same scenarios performed by three different pairs of actors yielded the similar affective responses and naturalness ratings. Likewise, we found high correlations (median *rS* = 0.95 *p* < 0.001, range for actor-pairs 0.95–0.96) on valence ratings of all videos belonging the same scenario performed by the three different actor-pairs, suggesting high inter-actor consistency for their performances. In the case of arousal (median *rS* = 0.44 *p* = 0.02, range 0.25–0.73) and naturalness ratings (median *rS* = 0.54 *p* = 0.01, range 0.48–0.67), we found weaker correlations among the three videos from the same scenarios. Based on the results, we could infer that participants perceived each scenario performed by three different actor-pairs rather similarly in all ratings (Fig 5C).

Importantly, the results from split-half reliability tests show a high degree of agreement on perceived pleasantness (*rSB1* = 0.99), arousal (*rSB1* = 0.97) and naturalness (*rSB1* = 0.94) of the social touch videos among participants, reaching to the ceiling (i.e. *rSB1* ≈ 1). We also observed high consistency when splitting the group based on the sex, indicating no sex differences in perceived valence (*rSB1* = 0.996), arousal (*rSB1* = 0.97) and naturalness (*rSB1* = 0.96) of the social touch communications.

#### Experiment 1: Affective responses to social stimuli

**Fig 6:**
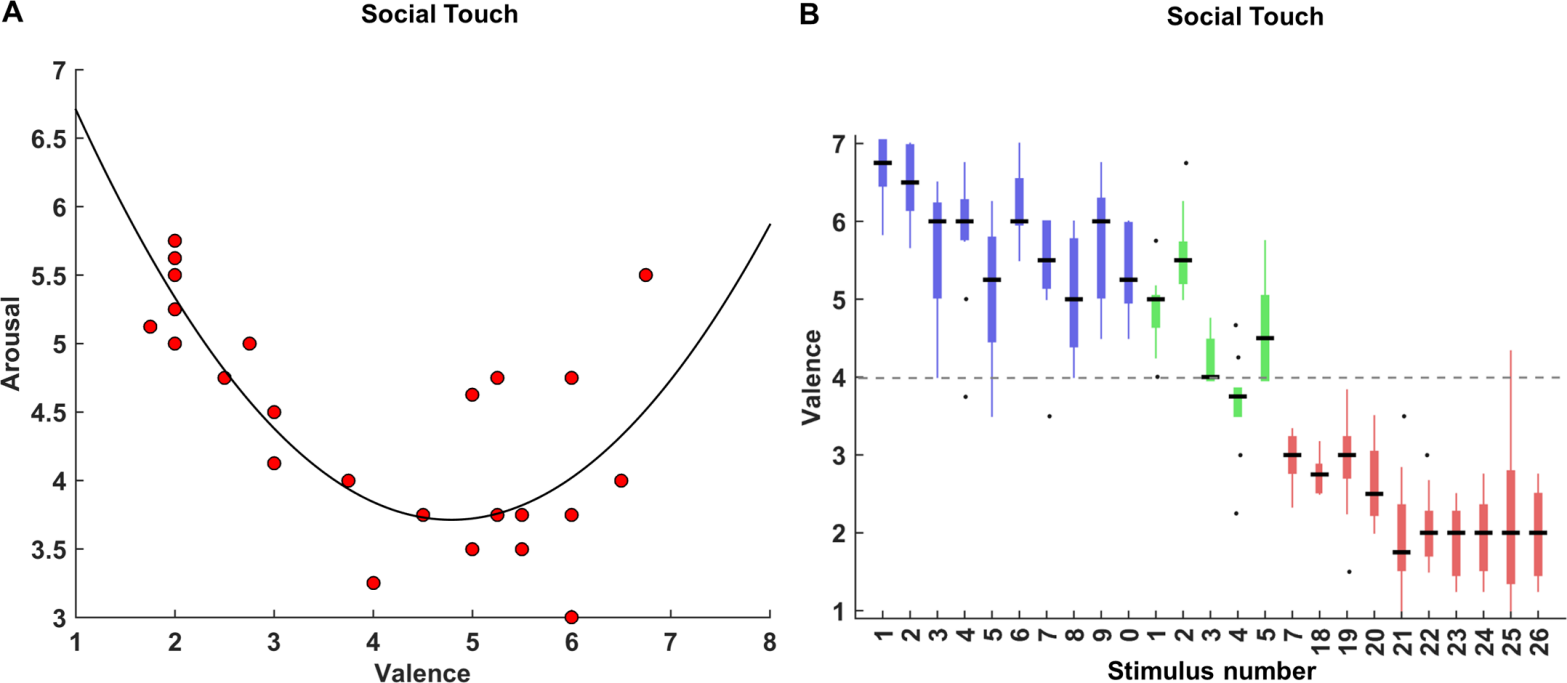
Affective responses to social stimuli. **(**A) The scatter plot and fitted quadratic function between group valence and arousal ratings of 25 scenarios present the typical U-shape distribution. The black curve indicates the fitted polynomial curve of degree 2 using a least-squares regression between valence and arousal. (B) The boxplots show individual valence ratings of 25 touch events performed by three actor-pairs. The central black lines indicate the group medians for each scenario. The top and bottom edges of the boxes illustrate the 75th and 25th percentiles respectively while the whiskers show the range of the individual valence ratings. The black dots indicate the outliers in (B). Different colours indicate the expected valence of each touch event [blue for the pleasant, green for the neutral and red for the unpleasant events]. Black dash line is dividing the stimuli between the pleasant and unpleasant touch events at the point of rating 4 (neutral valence), indicating the stimuli above the line are perceivably pleasantly while stimuli below the line are perceive unpleasantly.

We investigated the affective responses to the 25 scenarios. We were able to observe the typical U-shape relationship between arousal and valence [24] obtained from quadratic function fitting of the median averaged group ratings (Fig 6A). Importantly, all the positive touch events were perceived as pleasant (median = 6, MAD = 0.5) whereas the negative touch events were rated as unpleasant (median = 2, MAD = 0.13). The neutral events were mostly perceived neutrally (median = 4.5, MAD = 0.5). Thus, rated valence is significantly higher for the positive scenarios than the negative ones (*z* = 3.79, *p* < 0.001). Additionally, we also observed significant differences on the rated valence between positive scenarios and neutral scenarios (*z* = 2.54, *p* = 0.01) and negative scenarios and neutral scenarios (*z* = -3.06, *p* = 0.002) using the Mann-Whitney U test.” Fig 6B presents the individual valence ratings for 25 stimuli (See S1 Table for description of the stimuli).

#### Selection of a basic set of stimuli and object-based touch

Based on the results described above, we selected 6 positive, 1 neutral and 6 negative touch scenes as a basic set of stimuli (See Table 1, Fig 1). More specifically, our selection was made based on three factors: 1) naturalness of the touch scenes. Stimuli number 8 ((median naturalness = 4), 16 (median naturalness = 3.75), 21 (median naturalness = 4) and 22 (median naturalness = 4.25) were not selected since they were perceived *comparatively* less natural to other stimuli (see in Fig 3). 2) the match between expected and perceived valence of the stimuli. Based on the valence ratings, we did not select neutral touch scenes that were perceived as slightly positive (stimuli number 11 (median valence = 5) and 12 (median valence = 5.5)). 3) We considered the sex balances for both pleasant and unpleasant touch in order not to confound a specific sex with a specific affective value of the touch (e.g. men deliver only negative touch). More specifically, both sexes delivered equal amounts of pleasant (7 videos with males and 8 videos with females), neutral (2 videos with males and 1 video with females) and unpleasant touch (9 videos by each sex).

After the selection of the basic set of stimuli, we created the object-based non-social touch videos that had matched hand gestures with social videos such as hugging, stroking, and shaking. Moreover, we matched the actors between the social and object-based touch. The actors who delivered touch in each social scenario performed the matched object-based touch scene. We recorded a total of 36 videos [12 motion matched scene × 3 actor pairs]. It should be noted that one of the social videos where both actors are running to hug (See Table 1, stimulus name “Hug1_p”) could not be matched since an object cannot run and hug a person. Thus, a basic set of stimuli consists of 13 social and 12 object-based scenarios, performed by different actors.

#### Experiment 2: Affective responses to the basic set of social and object-based video clips

**Fig 7:**
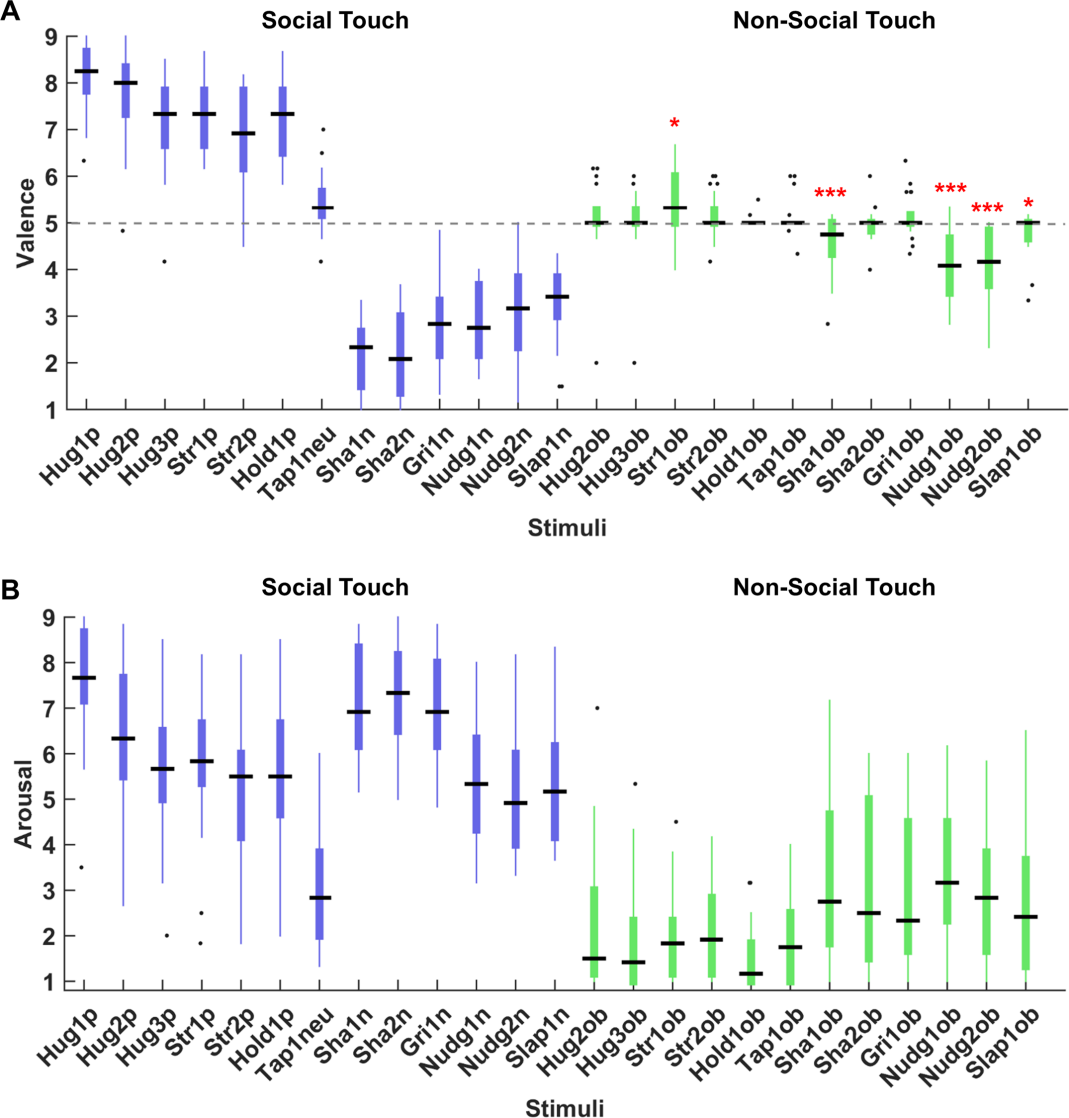
Affective responses to social and non-social stimuli. The boxplots show group valence (A) and arousal ratings (B) of 25 touch events performed by three actor-pairs for the social videos and 6 actors for the non-social videos. The central black lines indicate the group medians of individual ratings. The top and bottom edges of the boxes illustrate the 75th and 25th percentiles respectively while the whiskers show the range of the individual ratings. The black dots indicate the outliers in (A) and (B). Different colours indicate the distinction between social and non-social touch stimuli (blue for the social touch and green for the non-social touch events). Black dash line in the (A) is dividing the stimuli between the pleasant and unpleasant touch events at the point of rating 5 (neutral valence), indicating the stimuli above the line are perceived pleasantly while stimuli below the line are perceived unpleasantly. The single red asterisk indicates the statistical significance at *p* < 0.05, two red asterisks denotes statistical significance at *p* < 0.01, and three red asterisks show statistical significance at *p* < 0.001. X-axis indicates the name of stimuli (See Table 1).

We measured valence and arousal of each selected social touch video and its corresponding object-based touch video. Similar to the results described above (See Fig 6B), Positive touch events were perceived as pleasant (median = 7.33, MAD = 0.21) whereas negative events were perceived as unpleasant (median = 2.79, MAD = 0.42). Importantly, object-based touch events were rated as neutral (median = 5, MAD = 0.11). Fig 7 shows individual data for valence and arousal ratings of the 25 stimuli [13 social touch and 12 corresponding object-based touch], averaged across three video types and across two test sessions.

The Mann-Whitney U test revealed that rated valence was significantly higher for the positive scenarios than the negative ones (*z* = 5.14, *p* < 0.001). Additionally, we also observed the significant differences on the rated valence between positive scenarios and object-based touch scenarios (*z* = 6.36, *p* < 0.001) and negative scenarios and object-based touch scenarios (*z* = -6.29, *p* < 0.001).

The Wilcoxon sign rank test revealed that stimulus Str1_ob (*z* = 2.28, *p* = 0.02), Sha1_ob (*z* = -3.18, *p* = 0.001), Nudg1_ob (*z* = -3.84, *p* < 0.001), Nudg2_ob (*z* = -3.93, *p* < 0.001), and Slap1_ob (*z* = -2.11, *p* = 0.04) were significantly different from the neutral value 5 (See red asterisks in Fig 7A). Yet, all stimuli were rated significantly differently (*p* < 0.05) from the median ratings of positive (7.33) and negative touch (2.79), showing that object-based touch events were relatively neutral in terms of valence ratings. We also compared the rated arousal between social and non-social touch. The results revealed that social videos (except the neutral touch, stimulus “Tap1_neu”, see Fig 7B and Table 1) were rated as more arousing (median = 6, MAD = 0.97) than non-social videos (median = 2.13, MAD = 1.1) (*z* = 7.3, *p* < 0.001), as we intended.

Concerning the reliability of the full dataset, similar to Experiment 1, a high degree of inter-rater reliability was shown in perceived pleasantness (*rSB1* = 0.99) and arousal (*rSB1* = 0.99) of the videos when randomly splitting the total group of participants into two sub-groups. Similarly, the split-half coefficients for the valence and arousal ratings are very high when separating the group based on the sex, indicating no sex differences on perceived valence (*rSB1* = 0.99) and arousal (*rSB1* = 0.99) of the touch videos.

#### Motion energy and its association with affective responses

First, rank correlational analysis on motion energy across actor-pairs revealed that similar motion energies characterized the three pairs of actors for a social video set (13 videos per pair of actors) (median *rS* = 0.74, *p* = 0.006; range of the correlation values: 0.69–0.81) and an object-based video set (12 videos) (median *rS* = 0.62, *p* = 0.03; range: 0.46–0.67). Moreover, the results showed that motion energy between 36 social and 36 non-social videos are highly associated (*rS* = 0.66, *p* < 0.001) as we intended since the motions were matched between the social and non-social stimuli. In all cases, similar patterns of motion energy were used among actors during recordings (e.g. matched motion energy for three hugging scenes performed by three actor-pairs and matched motions between hugging somebody and carrying the box).

The Wilcoxon sign rank test was conducted to evaluate differences in motion energy between affective touch videos (N = 12, excluding the social scenario without an object match) and object-based touch videos (N = 12). We performed the test for each actor-pair. The results revealed that motion energy was not significantly different between social and object-based touch videos in two actor-pairs (*z* = 1.49, *p* = 0.13 in both cases) while one actor-pair showed a small difference between social and object-based touch videos (*z* = 2.12, *p* = 0.04). Overall, motion energy between social and non-social videos was therefore judged to be similar.

**Fig 8:**
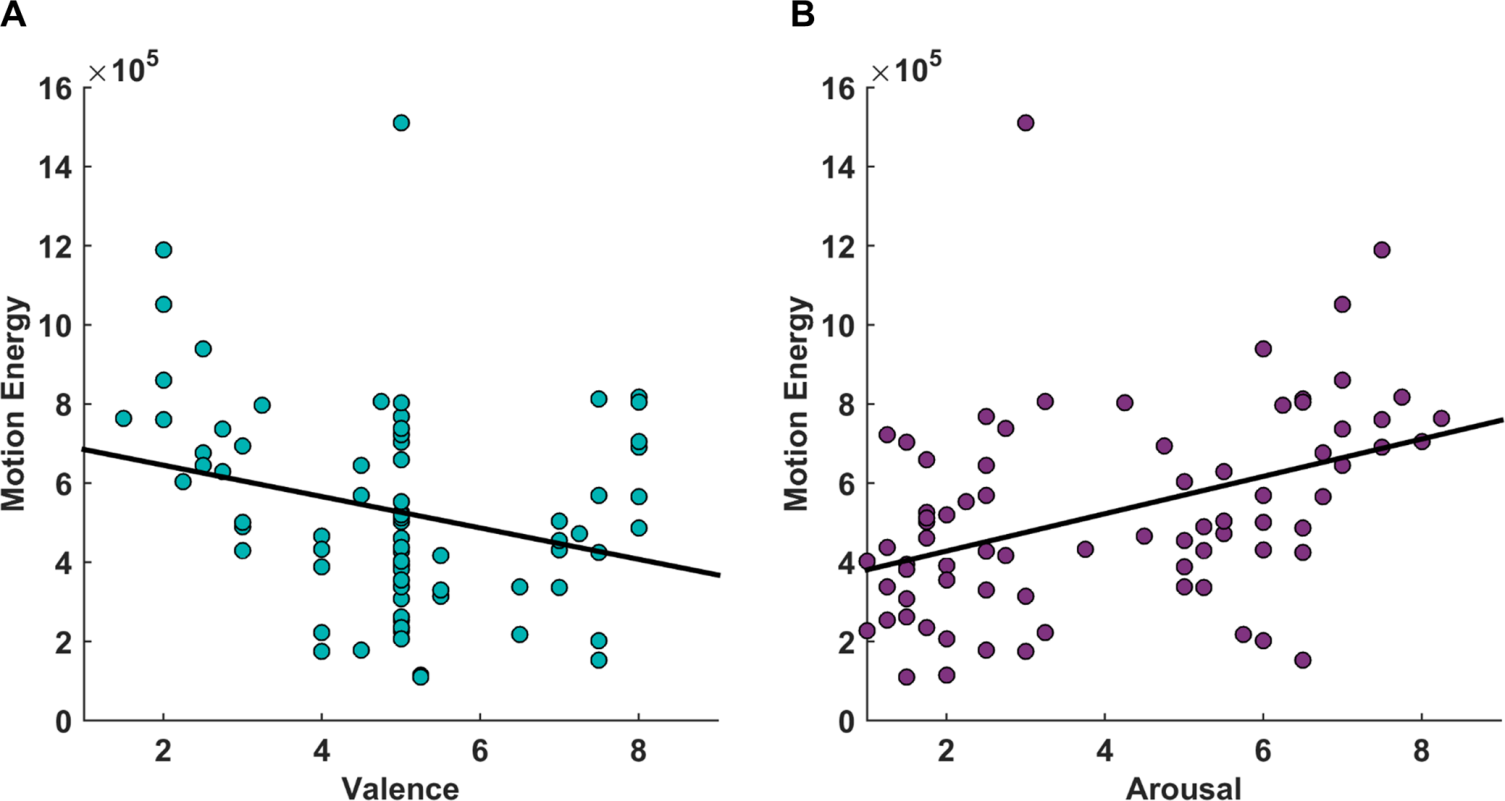
Motion energy and its association with affective responses. The scatter plots illustrate weak negative association between rated valence and motion energy (A) and moderate positive association between rated arousal and motion energy (B). Both x-axis represent 9-point Likert-like scale of ratings. The black trend lines in both plots indicate the linear relationship between the variables.

Lastly, correlational analysis between motion energy and affective ratings of all videos (N = 75) showed a significant, expected association between the rated arousal of touch events and their physical motion energy (*rS* = 0.47 [*rS* = 0.48 without the outlier], *p* < 0.001). Moreover, rated valence and motion energy also showed a weak association (*rS* = -0.27 [*rS* = -0.28 without the outlier], *p* = 0.02) (see Fig 8).

## Discussion

The aim of this study was to devise a reliable touch expression database that covers a large repertoire of interpersonal socio-affective touch. To achieve this goal, we created and validated a systematically defined, well-controlled socio-affective touch space, including negative, neutral, and positive situations with both low (calm) to high arousal (exciting) content. As non-social stimuli we also added video clips containing situations in which objects were touched without inducing specific emotions while matching each non-social action to a social touch event.

The results from the validation experiments enabled us 1) to eliminate artificially performed interpersonal touch scenes, 2) to measure whether interpersonal touch communication exhibits clear and replicable affective values and 3) to define intrinsic physical characteristics so as to control for or at least understand potentially unavoidable low-level physical parameters.

In spite of a large degree of individual variances in rank order according to naturalness of the social touch expressions, the majority of video clips (25 scenarios out of 26), performed by non-professional actors based on written scenarios, were perceived naturally. Although we did not provide either an utterly artificial or a highly natural touch expression as references before starting with the naturalness judgements task, based on the results that describe our participants being consistent between two repetitions, we conjecture that participant consistently relied on their own criteria for the judgements without varying them during the task, providing us with a useful exclusion criterion for the artificially performed touch scenes. Each touch expression performed by three different actor-pairs was also rated similarly on its naturalness, suggesting that there is a consistent difference between scenarios in terms of how easily the actors could perform them naturally. Indeed, informal discussions with the actors suggested that the negative scenarios were more difficult to perform; yet they were mostly perceived naturally. Given the results on rated naturalness, there is some ground to claim that the final socio-affective touch database (after the exclusion of some of the original videos) is composed of spontaneously performed, natural and life-like interpersonal touch expressions.

We also tested inter- and intra-observer consistencies on valence and arousal ratings. The results illustrated that participants were consistent on their ratings between two repetitions while conforming to others, showing both intra and inter-observer agreements on affective judgements. Moreover, rated affective values on each video clip performed by three different actor-pairs were also similar, indicating that our scenarios, used during recordings, successfully anchored the actor-pairs to perform in a way that positive touch events were perceived as pleasant, whereas negative touch events were perceived as unpleasant. When it comes to arousal, more variability within and across participants was found as compared to valence, which conforms with the findings of a previous study that investigated valence and arousal with stimuli from the frequently used International Affective Picture System [29]. Importantly, the typical U-shape relationship between arousal and valence was observed for our stimulus set [24], proving the validity and reliability of rated arousal with respect to valence. Overall, the results demonstrate that our socio-affective database elicit clear and replicable affective responses which span primary affective dimensions [20].

After selecting the basic set of stimuli, we created and validated corresponding object-based non-social touch stimuli. Here, our aim was to create object-based touch stimuli that did not evoke any emotion during observation. Indeed, our findings illustrate that object-based touch events are relatively neutral in terms of pleasantness as compared to social touch events. In the case of arousal, social touch turned out to be more arousing than object-based non-social touch, showing that interpersonal touch is emotionally more intense. It should be noted that the choice of which object to use in each object-based touch scenario is not definitive and various alternative objects could have been used if they are able to induce similar touch motion (e.g., the scenario showing a person carrying a box can be replaced by a scenario displaying a person carrying a large bag of rice). Furthermore, we admit that not every object-based touch scenario is a perfect match for social touch, since social touch events require more spontaneous interactions between two interacting people whereas an object cannot react when touched unless there is considerable inertia. Thus, some intrinsic variability in motions across actors and social versus object-based touch should be taken into account when using the current database.

Lastly, we systematically measured motion energy for all frames to characterize intrinsic physical properties of a particular action situation during social and non-social touch situation. Our findings revealed that the same video clips (e.g., hugging someone) acted by three different actor-pairs involved a similar amount of motion energy, indicating inter-actor consistency on performance for the given scenarios. Moreover, the same actions (e.g., hugging someone vs. hugging a box) were similarly characterized by this motion parameter regardless of socio-affective components embedded in the social touch situations. In a similar vein, total amount of motion energy consumed between social and non-social videos does not differ, implying inter-action consistency on consumed motion energy.

We also observed a positive association between rated arousal and motion energy in line with the expectation based on previous findings that demonstrated high motion energy in emotionally charged movie scenes displaying anger, horror and excitement, implying an intrinsic link between the two [30, 31].

Although our database, SATED, carries an unprecedented large range of dynamic interpersonal touch communication, we should admit that it still leaves out other parts of affective touch events. First, we selected between-sex social touch interaction scenarios for which we expected very little ambiguity in valence given the customs in Belgium. In a similar vein, we did not include same-sex social touch scenarios in the database due to foreseen high variance in affective touch response across observers [32]. Thus, when using the database, other researchers should consider the cultural from which participants are selected. Second, real-life touch events regularly involve a face (e.g., stroking or slapping a face). Yet, there are various reasons for why the faces should be excluded in the touch database. Mainly, we allowed actors to talk and make facial expressions freely during acting in order to ensure life-like naturalness. Thus, revealing the faces may be distracting due to facial movements or speech. Moreover, observers may rely more on facial expressions when judging the affective meaning of touch since the emotional state of the scene can be decoded by observing facial expressions [33–40]. Additionally, revealing the facial identity of actors could modulate the affective judgements on touch communication due to face attractiveness. Based on a previous study, attractiveness of a touch communicator can influence perceived pleasantness of affective touch [41]. Thus, in this database, we controlled for this variable by limiting the touch information to the torso region without any face information, and by all actors wearing similar clothes (See Methods).

It is worth mentioning that not every motion performed by different actors/actor-pairs was perfectly matched. Yet, humans are good at decoding emotions and intentions when observing human body movements even in the presence of significant degradation of presented information, such as a stimulus made of a few point-lights imitating human body movements [42]. Thus, we focused on matching the global motions, presented emotions and intentions in three videos performed by the different actors/actor-pairs. Based on this, the subtle differences shown in the videos (such as the number of times a bell is rung for attention or the angle at which actors are positioned) will not present limitations to the usefulness of the current database.

Lastly, although the ratings from participants were highly consistent across valence, arousal and naturalness judgements, it should be acknowledged that the current study has a relatively small sample size. Thus, our results should be interpreted with caution when generalizing the current findings to different population groups such as investigating effects of age or cultural background. In a similar vein, although we found no indication of sex differences in affective touch response, it should be noted that investigating sex differences was not a major aim of the current study and a separate study should be conducted with larger sample size to further investigate sex differences in ratings.

In summary, we created a large interpersonal touch expression database, the Socio-Affective Touch Expression Database (SATED), containing both social and non-social events. This validated database can facilitate research on human touch communication, serving as an advanced set of stimuli, ultimately enabling us to investigate complex interpersonal touch communication. We envision that our database can be used in various domains such as affective computing and social neuroscience.

Our database, SATED, is freely available in Open Science Framework (https://osf.io/8j74m/) for scientific use.

## Supporting information

S1 Fig Intra-subject consistency. This schematic figure illustrates a set of steps involved in data analysis per subject for Test A. The coloured columns (red, green and blue) indicate three different actor-pairs (A-B, C-D and E-F pairs) who performed each scenario. The row names such as “Hug” and “Pat” indicate touch expressions displayed in each stimulus. Each participant rated each video once per session for three scales (valence, arousal and naturalness). The analysis process for every scale is the same. The three ratings were averaged across every row in the first step (1: Average), followed by correlating the ratings from the first session with the ones from the second session (2: Correlation). Note that only 4 rows are displayed per session and per actor-pair instead of 25 for convenience.
(TIF)

S2 Fig Inter-subject consistency. This schematic figure illustrates a set of steps involved in data analysis for Test B. The ratings shown in the first step (1: Average) were averaged across every row, yielding two rating columns (one column per session) per participant. Then, during the second step (2: Average), the two rating columns from two sessions were averaged. The white columns shown in the third step (3: Pairwise correlations) indicate sets of ratings from the participants. Note that only 3 participants (shown in white columns in the third step) are displayed instead of 11 for convenience.
(TIF)

S3 Fig Inter-actor consistency. This schematic figure illustrates a set of steps involved in data analysis for Test C. The first step (1: Average) illustrates that the individual ratings from two sessions were averaged, resulting in three rating columns for each participant. The individual ratings per column were then averaged, resulted in total three rating columns (2: Group Average). Lastly, the third step (3: Pairwise correlations) was performed. Again, note that only 4 stimuli are displayed per session and per actor-pair instead of 25 for convenience.
(TIF)

S1 Table Scenarios of the full set of touch events. This table lists all the scenarios used for the recording of interpersonal touch expressions (both selected as a basic set of stimuli and an extended set of stimuli). Video number illustrates the file name of each video uploaded (extended socio-affective touch version). The expected valence and action for each touch expression are described. It should be noted that these videos should be used with caution since some of them did not pass the test on naturalness judgements. Moreover, the video numbers does not match with the one from the basic set of stimuli.
(DOCX)
