# Supplementary figures and images for: Socio-affective touch expression database
Source: PLoS One. 2018 Jan 24;13(1):e0190921. doi: 10.1371/journal.pone.0190921 (PMC5783378; doi:10.1371/journal.pone.0190921)

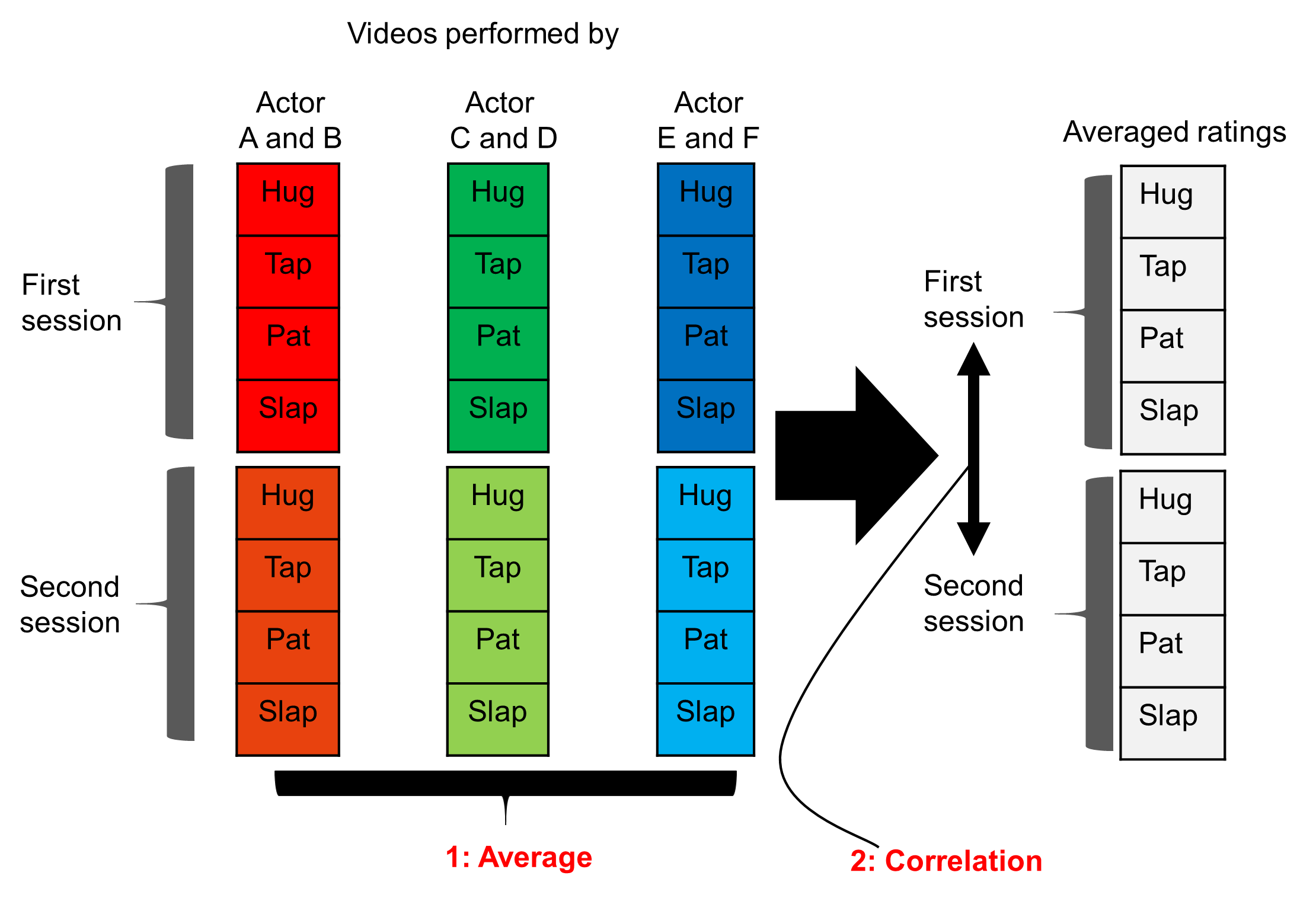

Supplement: S1 Fig — This schematic figure illustrates a set of steps involved in data analysis per subject for Test A. The coloured columns (red, green and blue) indicate three different actor-pairs (A-B, C-D and E-F pairs) who performed each scenario. The row names such as “Hug” and “Pat” indicate touch expressions displayed in each stimulus. Each participant rated each video once per session for three scales (valence, arousal and naturalness). The analysis process for every scale is the same. The three ratings were averaged across every row in the first step (1: Average), followed by correlating the ratings from the first session with the ones from the second session (2: Correlation). Note that only 4 rows are displayed per session and per actor-pair instead of 25 for convenience. (TIF) [file pone.0190921.s001.tif]

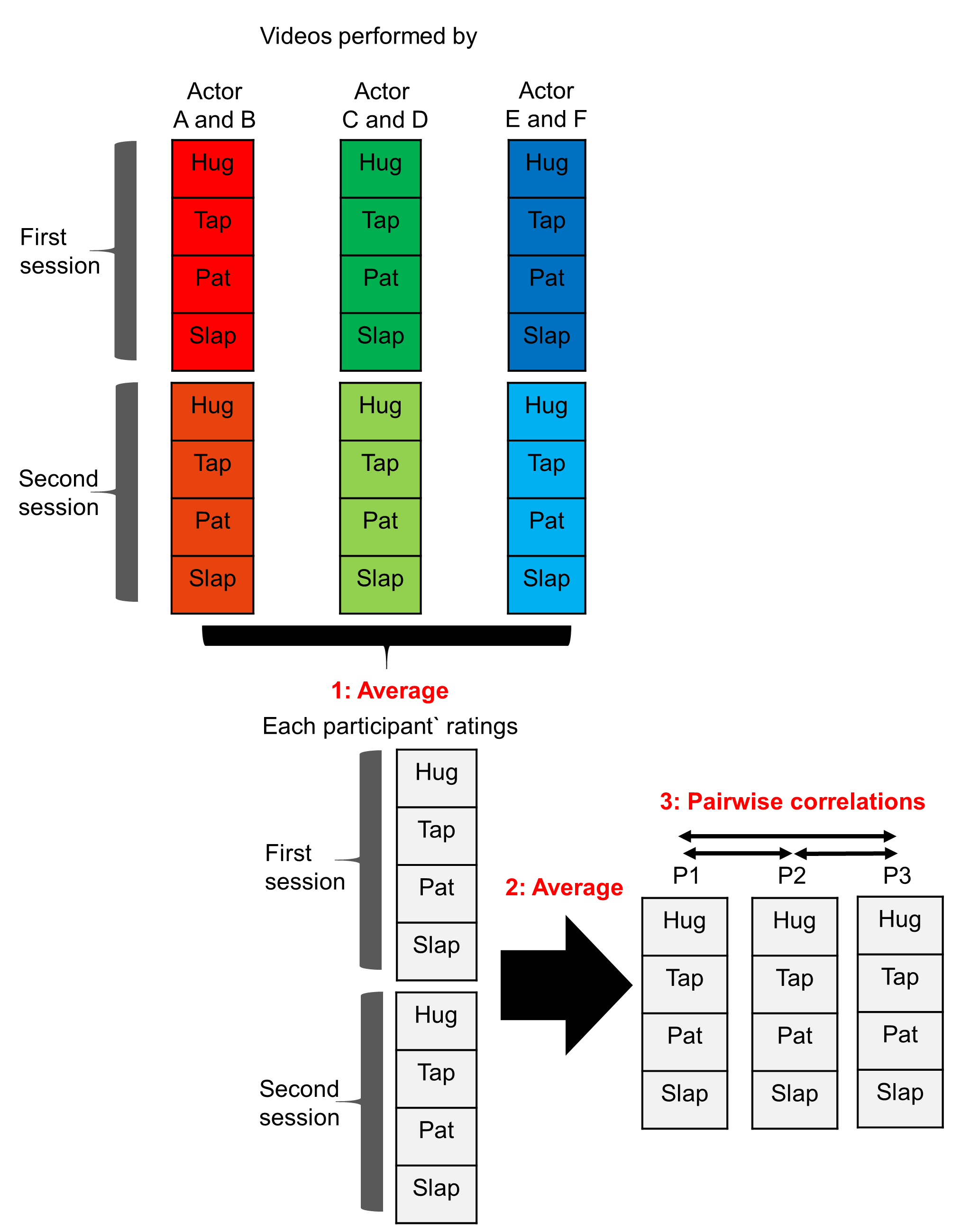

Supplement: S2 Fig — This schematic figure illustrates a set of steps involved in data analysis for Test B. The ratings shown in the first step (1: Average) were averaged across every row, yielding two rating columns (one column per session) per participant. Then, during the second step (2: Average), the two rating columns from two sessions were averaged. The white columns shown in the third step (3: Pairwise correlations) indicate sets of ratings from the participants. Note that only 3 participants (shown in white columns in the third step) are displayed instead of 11 for convenience. (TIF) [file pone.0190921.s002.tif]

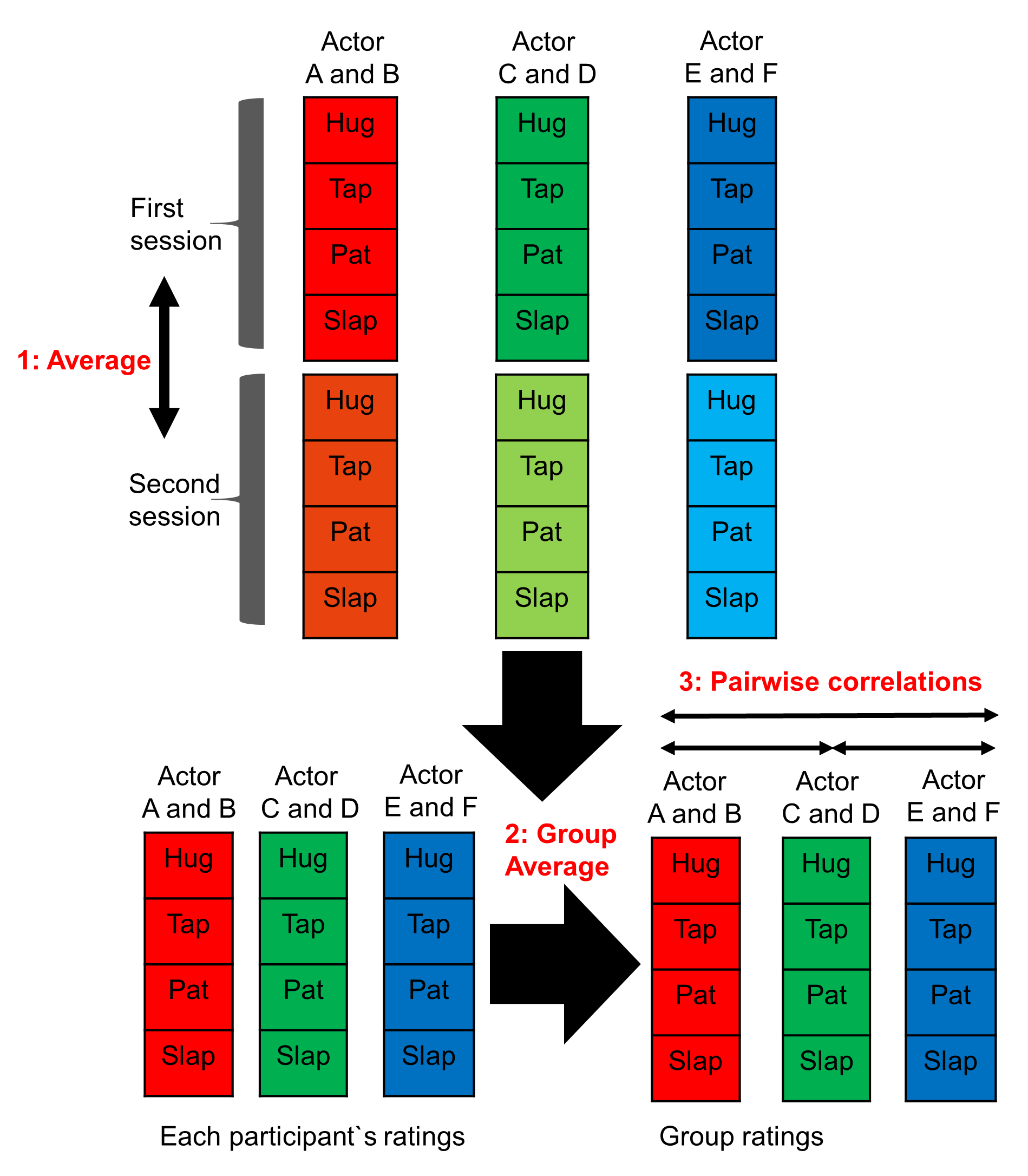

Supplement: S3 Fig — This schematic figure illustrates a set of steps involved in data analysis for Test C. The first step (1: Average) illustrates that the individual ratings from two sessions were averaged, resulting in three rating columns for each participant. The individual ratings per column were then averaged, resulted in total three rating columns (2: Group Average). Lastly, the third step (3: Pairwise correlations) was performed. Again, note that only 4 stimuli are displayed per session and per actor-pair instead of 25 for convenience. (TIF) [file pone.0190921.s003.tif]
