# Supplementary material for: Socio-affective touch expression database
Source: PLoS One. 2018 Jan 24;13(1):e0190921. doi: 10.1371/journal.pone.0190921 (PMC5783378; doi:10.1371/journal.pone.0190921)
Supplement: S1 Table — This table lists all the scenarios used for the recording of interpersonal touch expressions (both selected as a basic set of stimuli and an extended set of stimuli). Video number illustrates the file name of each video uploaded (extended socio-affective touch version). The expected valence and action for each touch expression are described. It should be noted that these videos should be used with caution since some of them did not pass the test on naturalness judgements. Moreover, the video numbers does not match with the one from the basic set of stimuli. (DOCX) [file pone.0190921.s004.docx]

| Video Number | Scenario | Valence | Action |
| --- | --- | --- | --- |
| 1-1, 2-1, 3-1 | You are in the airport. You and your partner have not seen each other for 6 month. You hug each other as soon as you see each other. | Positive | Hug |
| 1-2, 2-2, 3-2 | Your partner looks somehow lovelier today. You want to give a hug to express how much you love this person. | Positive | Hug |
| 1-3, 2-3, 3-3 | Your friend got promoted at work. You congratulate him (her) on his (her) promotion whiling showing how much you are proud of him (her) by hugging. | Positive | Hug |
| 1-4, 2-4, 3-4 | Your sibling just ended the long-term relationship. You want to console him (her) by hugging. | Positive | Hug |
| 1-5, 2-5, 3-5 | Your partner is highly frightened. You want to make this person ensure he (she) is safe now by hugging. | Positive | Hug |
| 1-6, 2-6, 3-6 | You want to flirt with him (her) by stroking his (her) arm with intimacy. | Positive | Stroke |
| 1-7, 2-7, 3-7 | Your friend is crying. You want to console this person by stroking on his (her) arms. | Positive | Stroke |
| 1-8, 2-8, 3-8 | You just heard that you two are both accepted in the same university. You hold hands and shake with joy. | Positive | Hold |
| 1-9, 2-9, 3-9 | You hold hands of your partner with affection. | Positive | Hold |
| 1-10, 2-10, 3-10 | You do a fist bump with your friend as a cool greeting or a way to celebrate small thing. | Positive | Fist bump |
| 1-11, 2-11, 3-11 | You meet this person for the first time. You want to greet by shaking hands. | Neutral | Hold |
| 1-12, 2-12, 3-12 | The sibling of your partner (who you don't know well) visited your house. You open the door and welcome them by hugging only as a greeting purpose. | Neutral | Hug |
| 1-13, 2-13, 3-13 | You want to get an attention from your colleague (who could not hear you calling) by tapping his (her) shoulder. | Neutral | Tap |
| 1-14, 2-14, 3-14 | You are the senior. You are talking to your junior student “Let’s move” while guiding them to certain direction by touch. | Neutral | Pat |
| 1-15, 2-15, 3-15 | Your colleague is sleeping during the meeting. You want to wake him (her) up by nudging in his (her) arm. | Neutral | Nudge with elbow |
| 1-16, 2-16, 3-16 | You work at the airport. You need to check if this person has a weapon in the pocket or underneath of the clothes. | Neutral | Pat |
| 1-17, 2-17, 3-17 | You are in the metro. You need to get off from the metro. There is somebody blocking you. You nudge your way by slightly removing somebody’s arm blocking you. | Negative | Nudge with elbow |
| 1-18, 2-18, 3-18 | You are so in a hurry. You don't care if other people will perceive you rude or not. You just shove other person’s shoulder (arm) to pass through. | Negative | Push with hands |
| 1-19, 2-19, 3-19 | Your sibling tries to be silly by making a weird gesture. You are annoyed. You want to stop him (her) by poking in the ribs. | Negative | Nudge with elbow |
| 1-20, 2-20, 3-20 | Your sibling always makes the same mistakes. You slap his (her) arm to make him (her) realize that you are annoyed and that you expect something better. | Negative | Slap |
| 1-21, 2-21, 3-21 | Your car collided at an intersection with other car. During the argument, the driver of the other car kicked your car with his (her) foot. You want to show how angry you are to stop him (her) by punching his (her) arm. | Negative | Punch |
| 1-22, 2-22, 3-22 | Your neighbour often plays the trumpet at night. You cannot sleep with this sound. You are now in front of his (her) house to discuss. He (She) does not want to listen to you. Instead, he (she) aggressively gets too close to your face during the conversation. You want to push him (her) away. | Negative | Push |
| 1-23, 2-23, 3-23 | You just found out that your partner cheated on you. You are very disappointed and angry. You ask him (her) how and why this happened aggressively while shaking his (her) whole body. | Negative | Shake |
| 1-24, 2-24, 3-24 | Your sibling committed crime. You are sad and angry since you cannot understand why he (she) made such a horrible decision. You firmly ask him (her) how and why this has happened while shaking his (her) arm. | Negative | Shake |
| 1-25,2-25,3-25 | Your partner said that you two are over from now on. He or she is about to walk away and never see you again. You cannot let this person go. You cannot let this person go. You hug this person’s body forcefully and desperately. | Negative | Hug |
| 1-26, 2-26, 3-26 | Your partner said that you two are over from now on. He or she is about to walk away and never see you again. You cannot let this person go. You cannot let this person go. You grip this person’s arm desperately. | Negative | Grip |
